# Supplementary material for: Design, Synthesis, and Molecular Docking Study of New Tyrosyl-DNA Phosphodiesterase 1 (TDP1) Inhibitors Combining Resin Acids and Adamantane Moieties
Source: Pharmaceuticals (Basel). 2021 May 1;14(5):422. doi: 10.3390/ph14050422 (PMC8147275; doi:10.3390/ph14050422)
Supplement: Supplementary file 1 [file pharmaceuticals-14-00422-s001.zip › pharmaceuticals-1179233-supplementary.pdf]

^{1}H and ^{13}C NMR spectra for compound 2

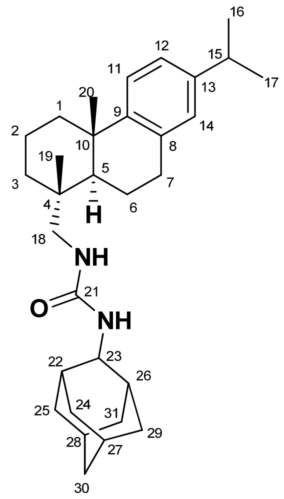

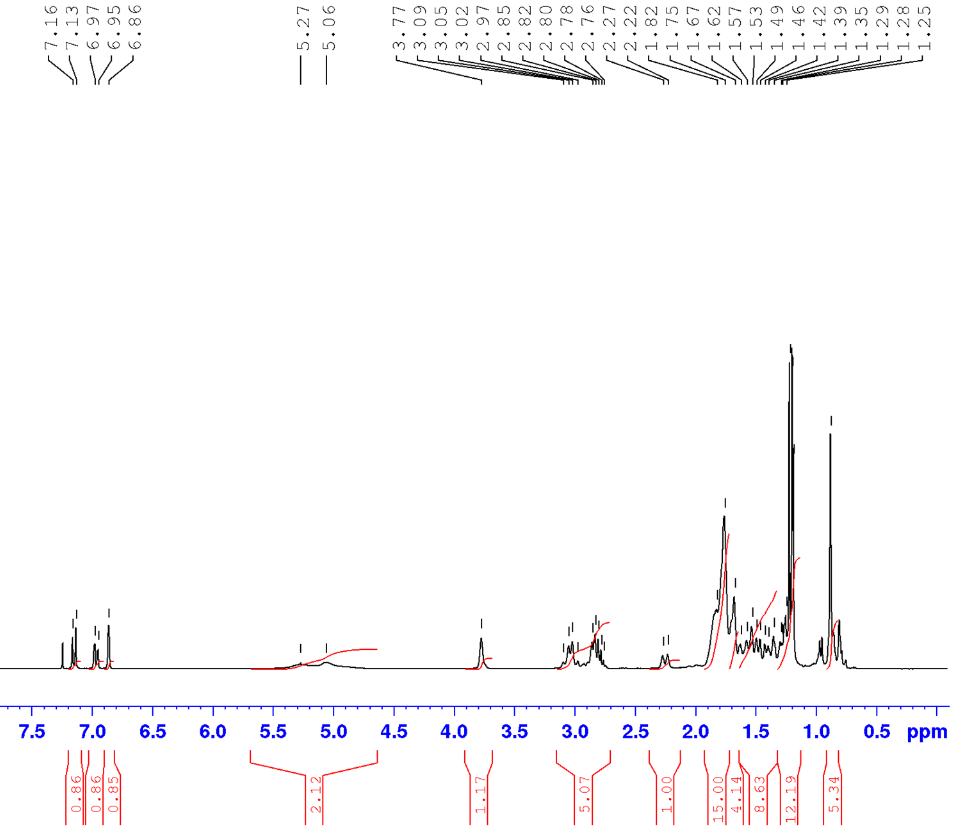

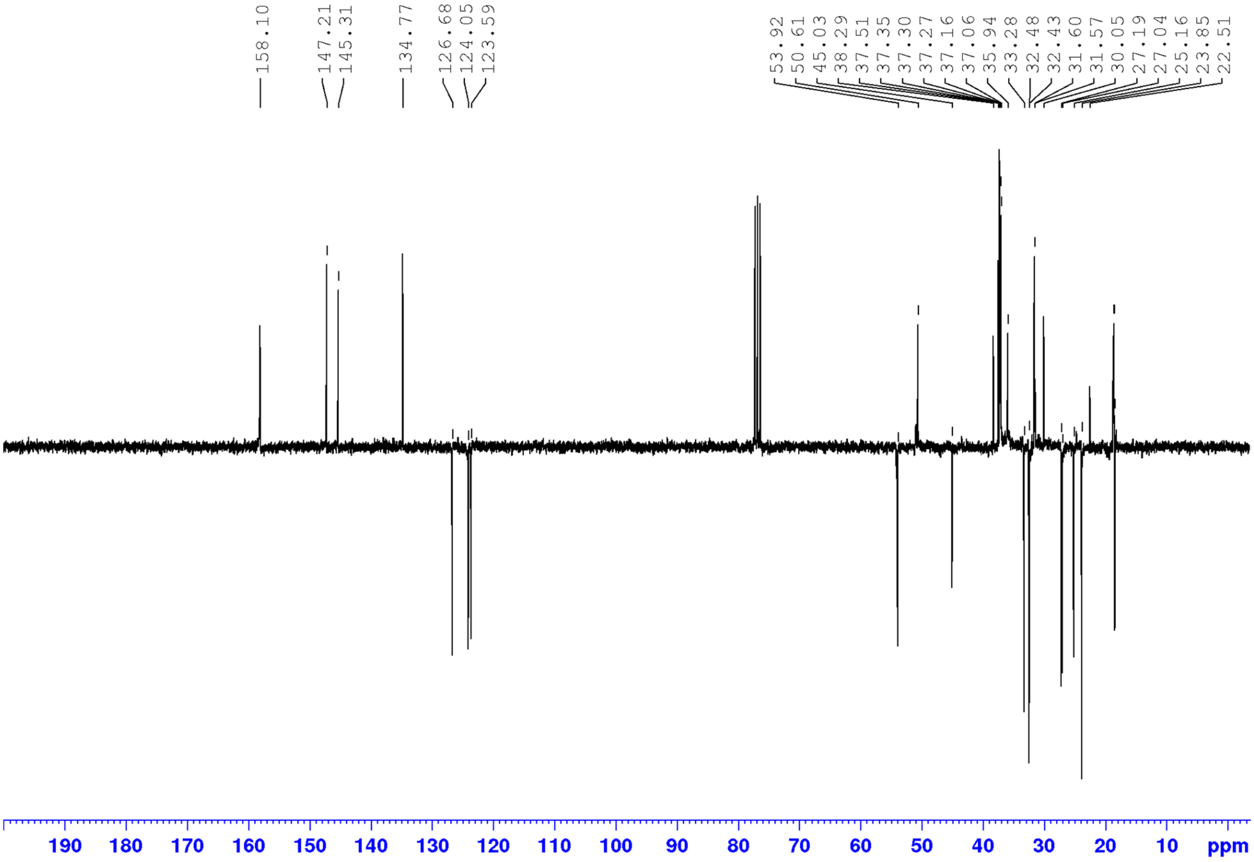

^{1}H and ^{13}C NMR spectra for compound 3

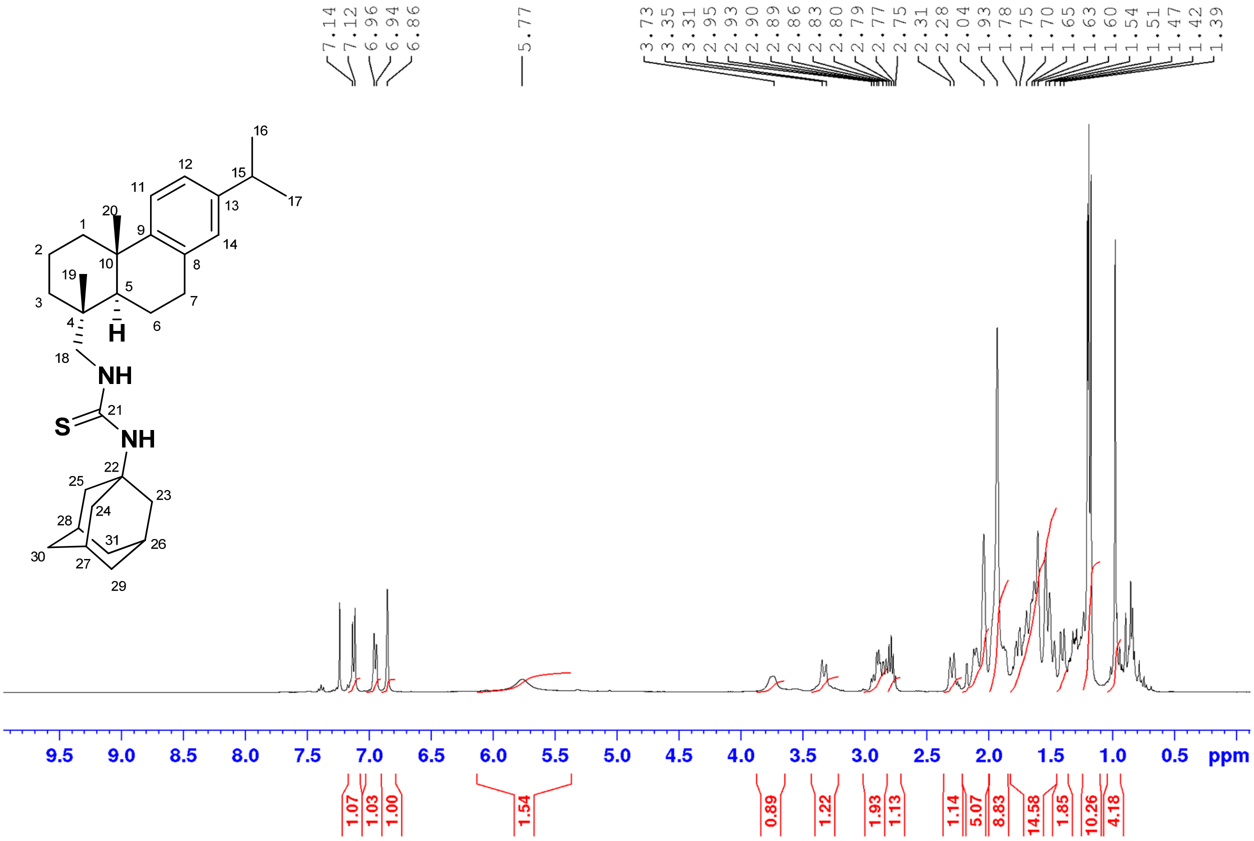

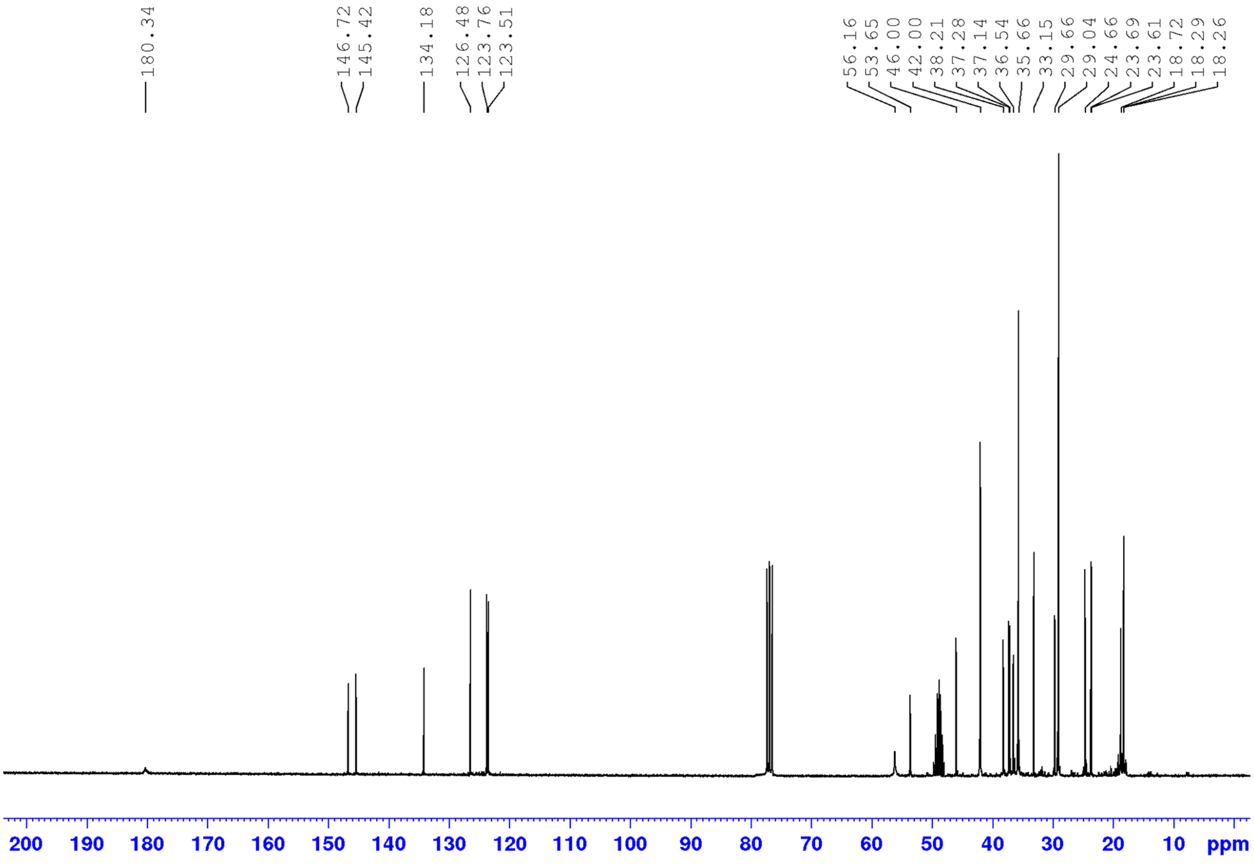

^1H and ^{13}C NMR spectra for compound 4

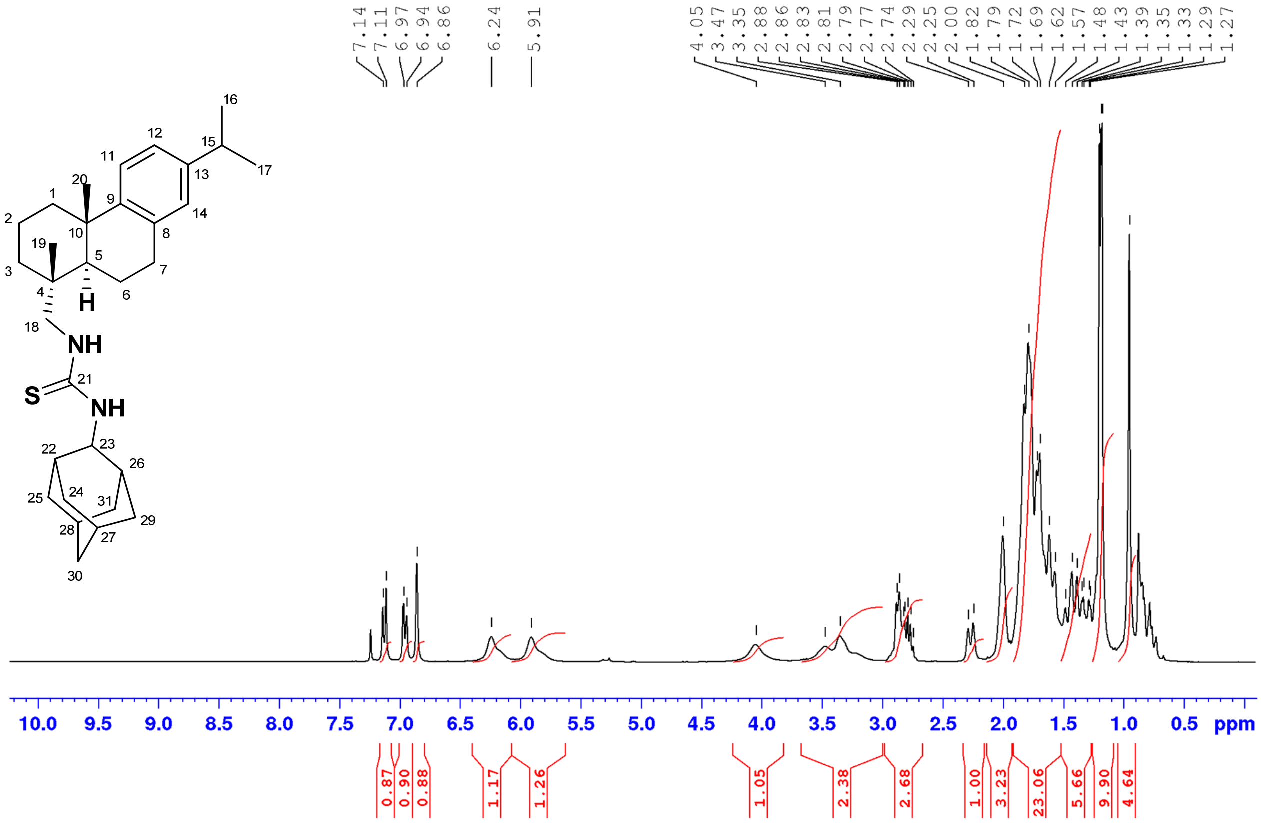

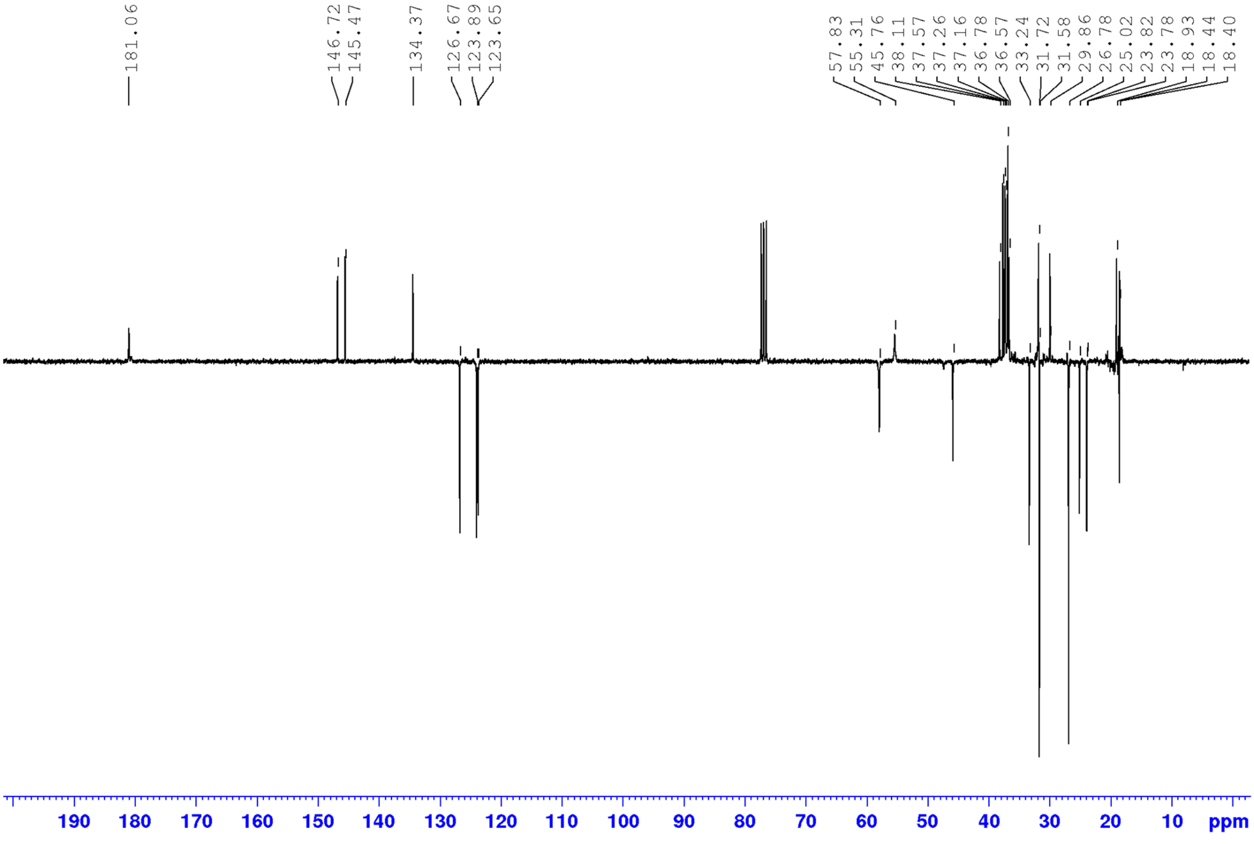

^{1}H and ^{13}C NMR spectra for compound 5

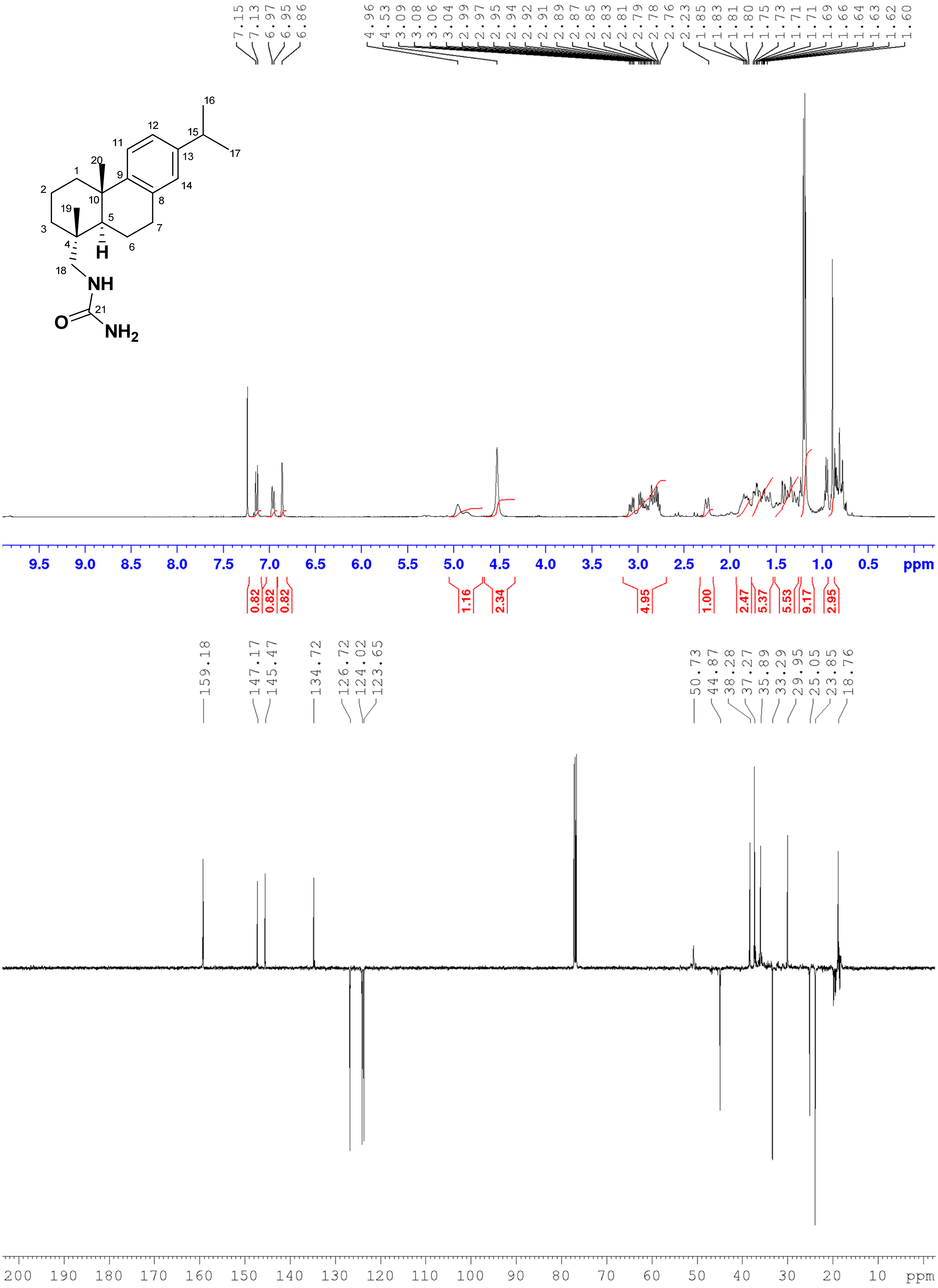

^{1}H and ^{13}C NMR spectra for compound 6

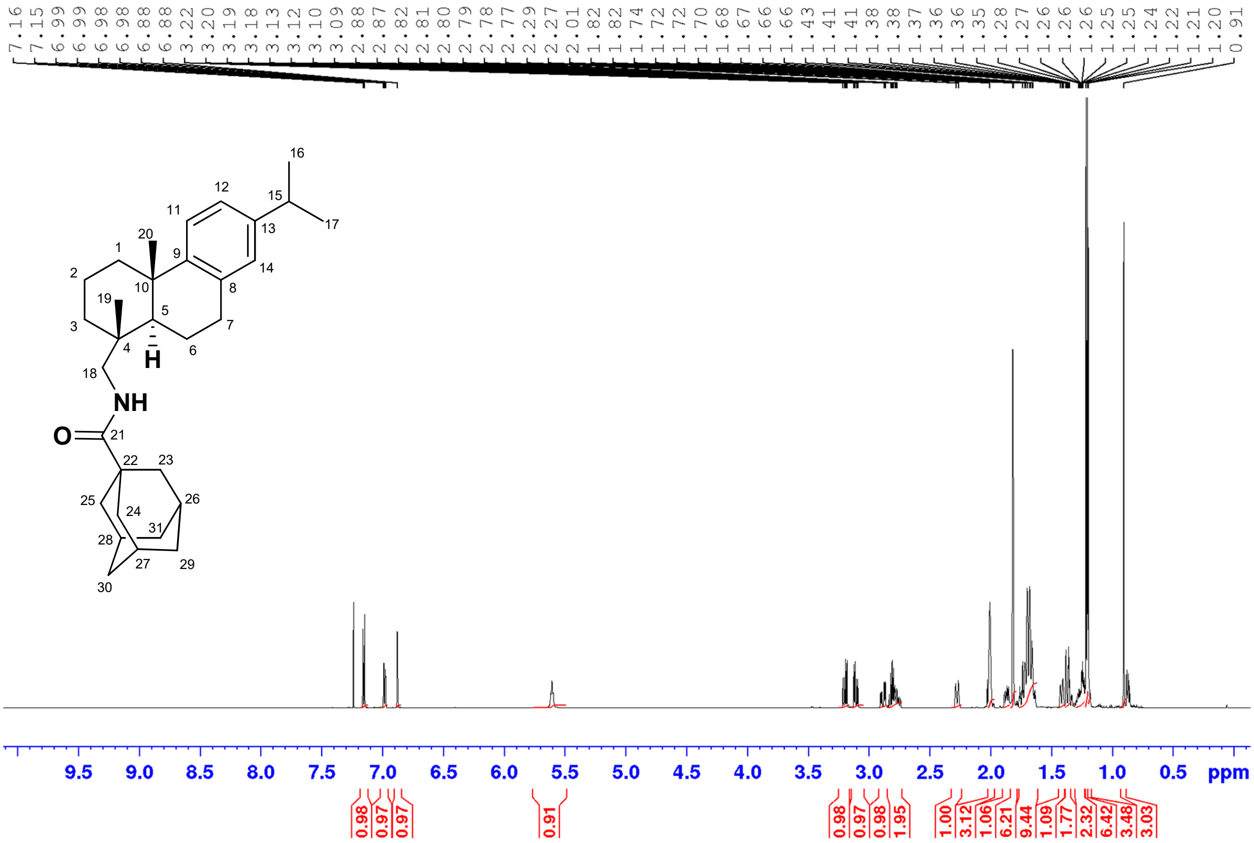

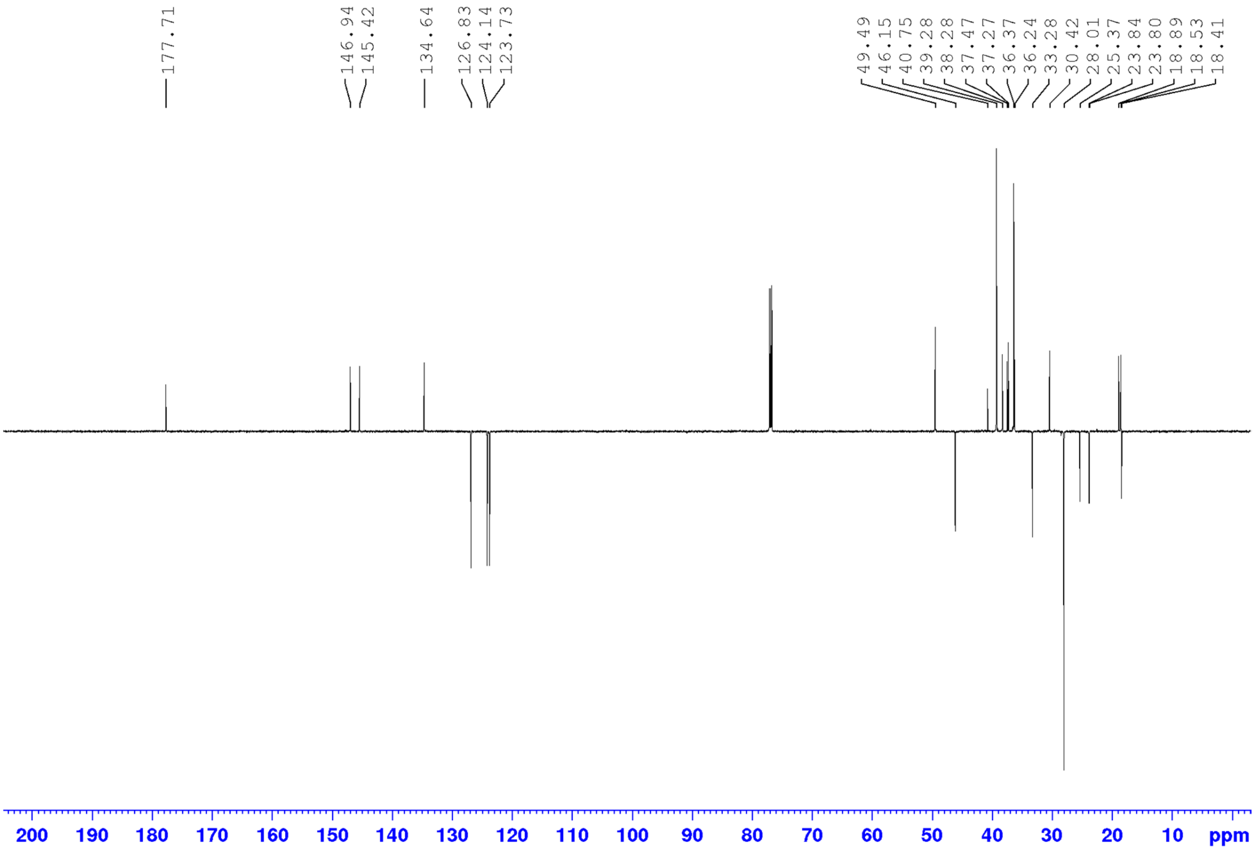

^{1}H and ^{13}C NMR spectra for compound 7

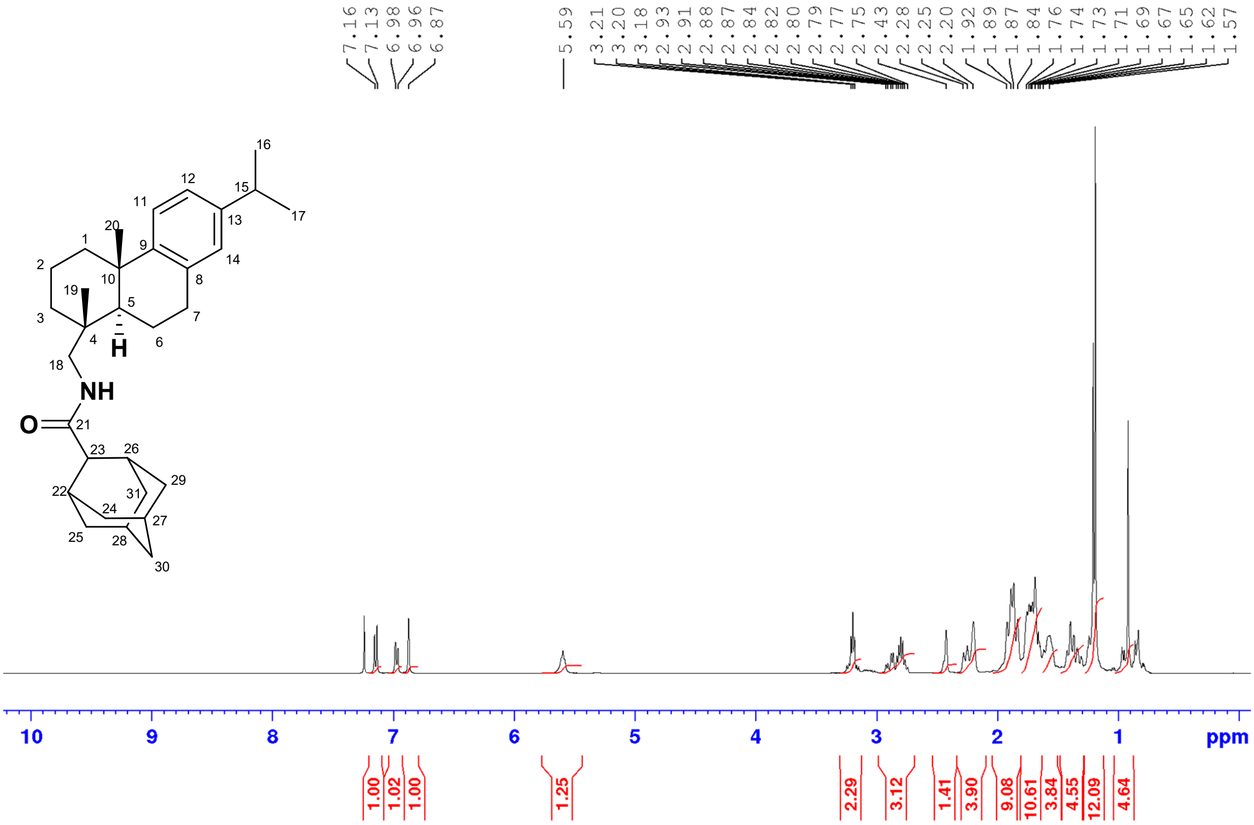

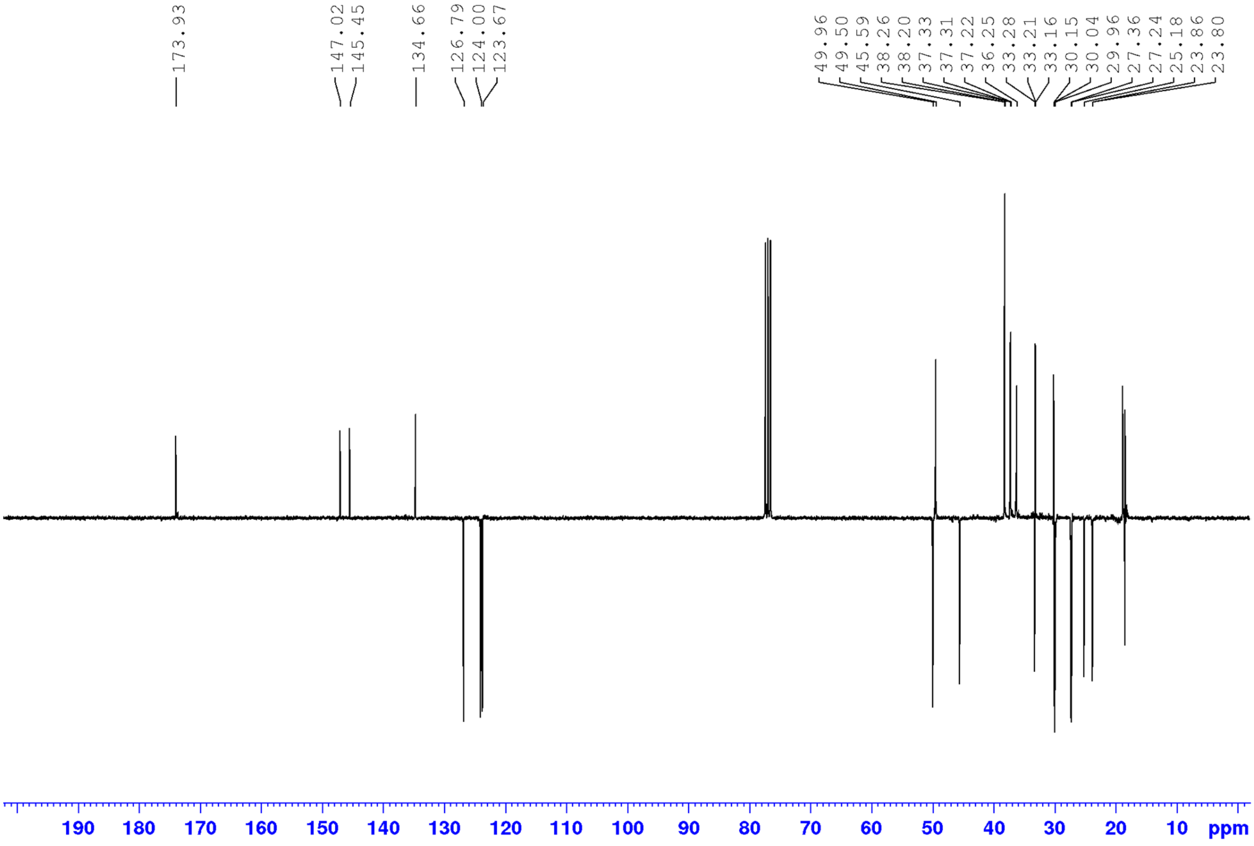

^{1}H and ^{13}C NMR spectra for compound 8

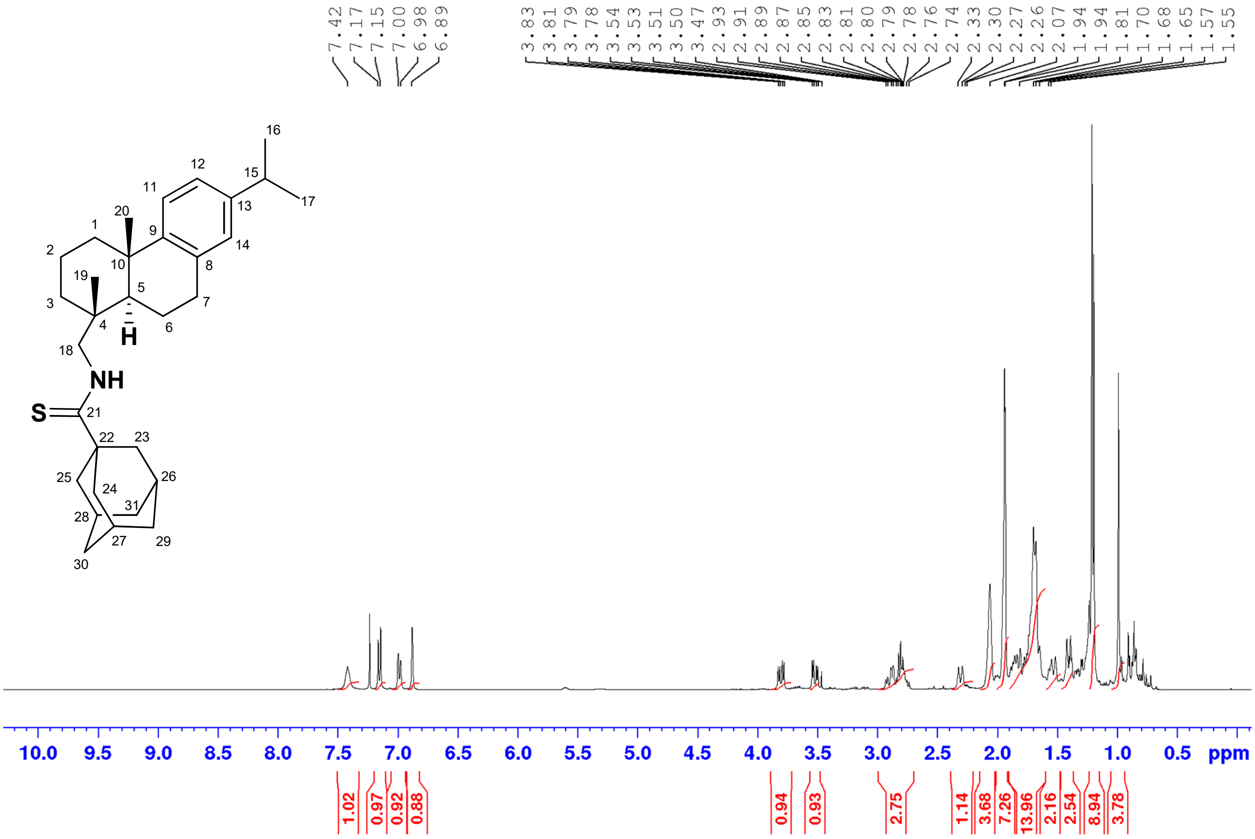

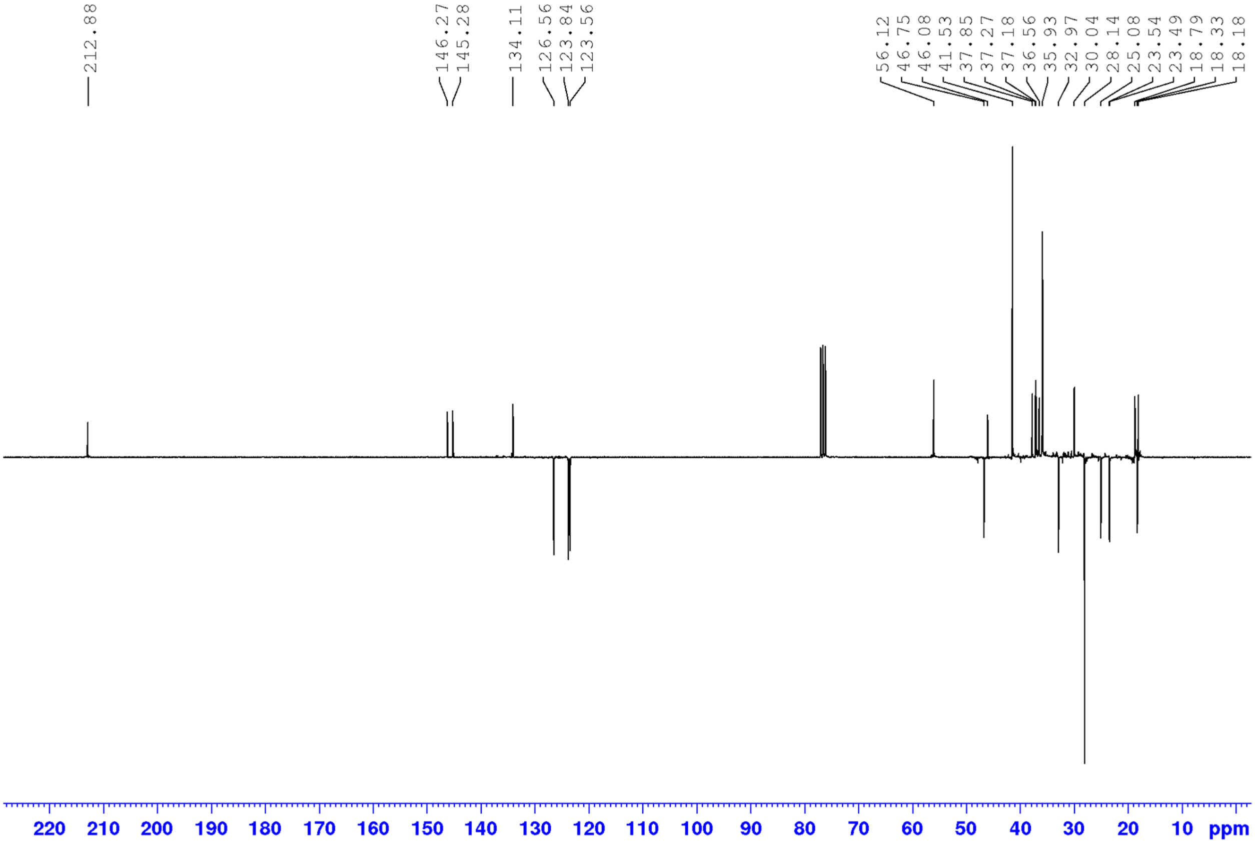

^{1}H and ^{13}C NMR spectra for norabietyl isocyanate

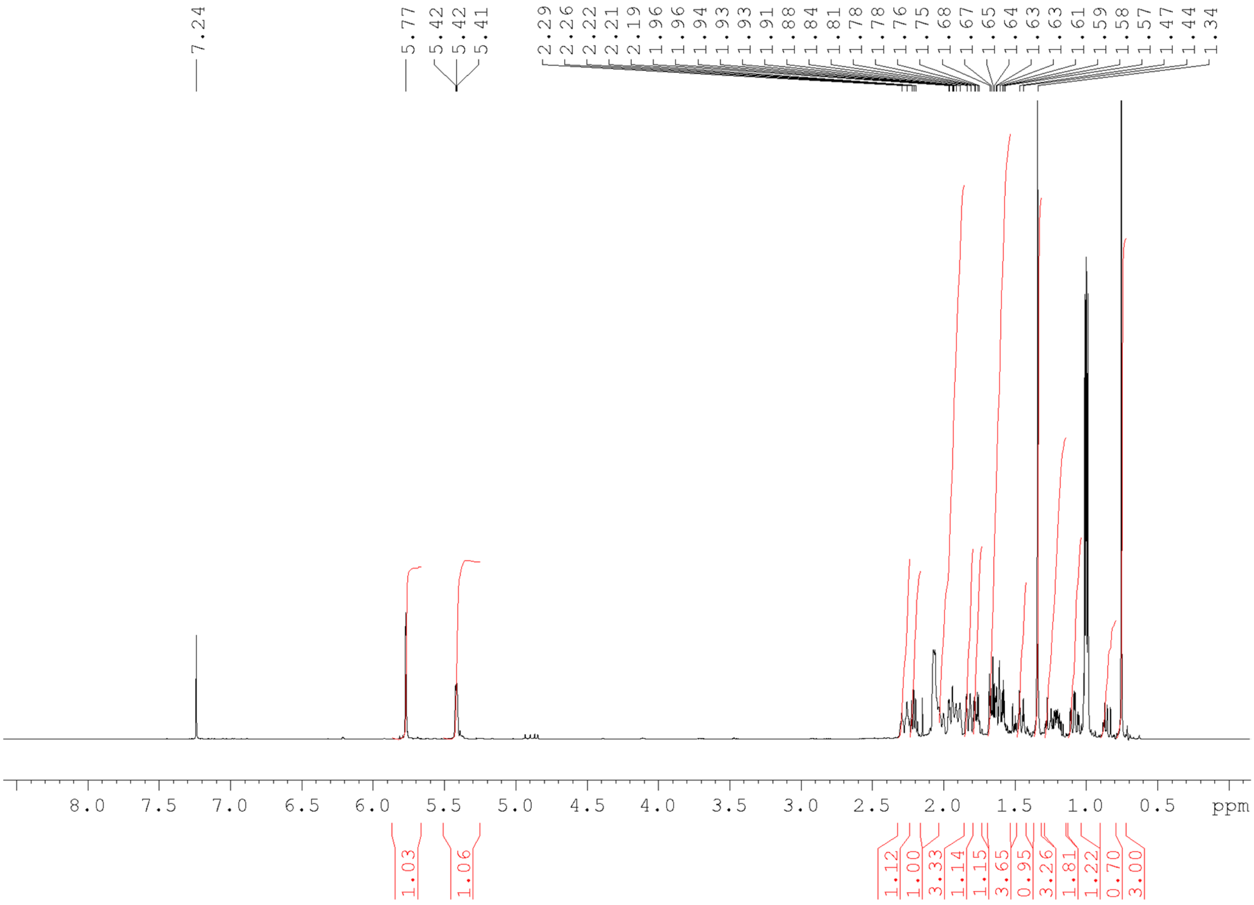

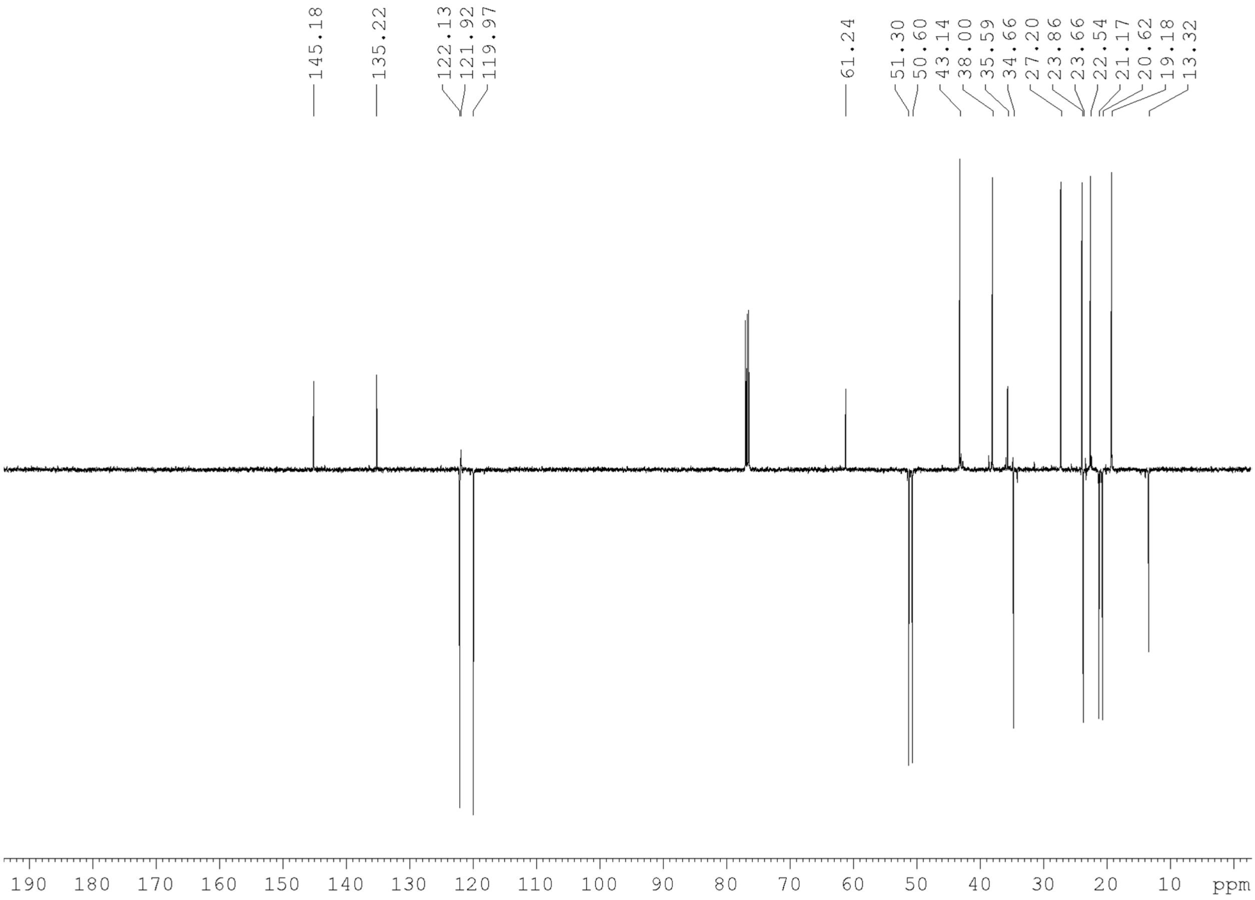

^{1}H and ^{13}C NMR spectra for compound 9

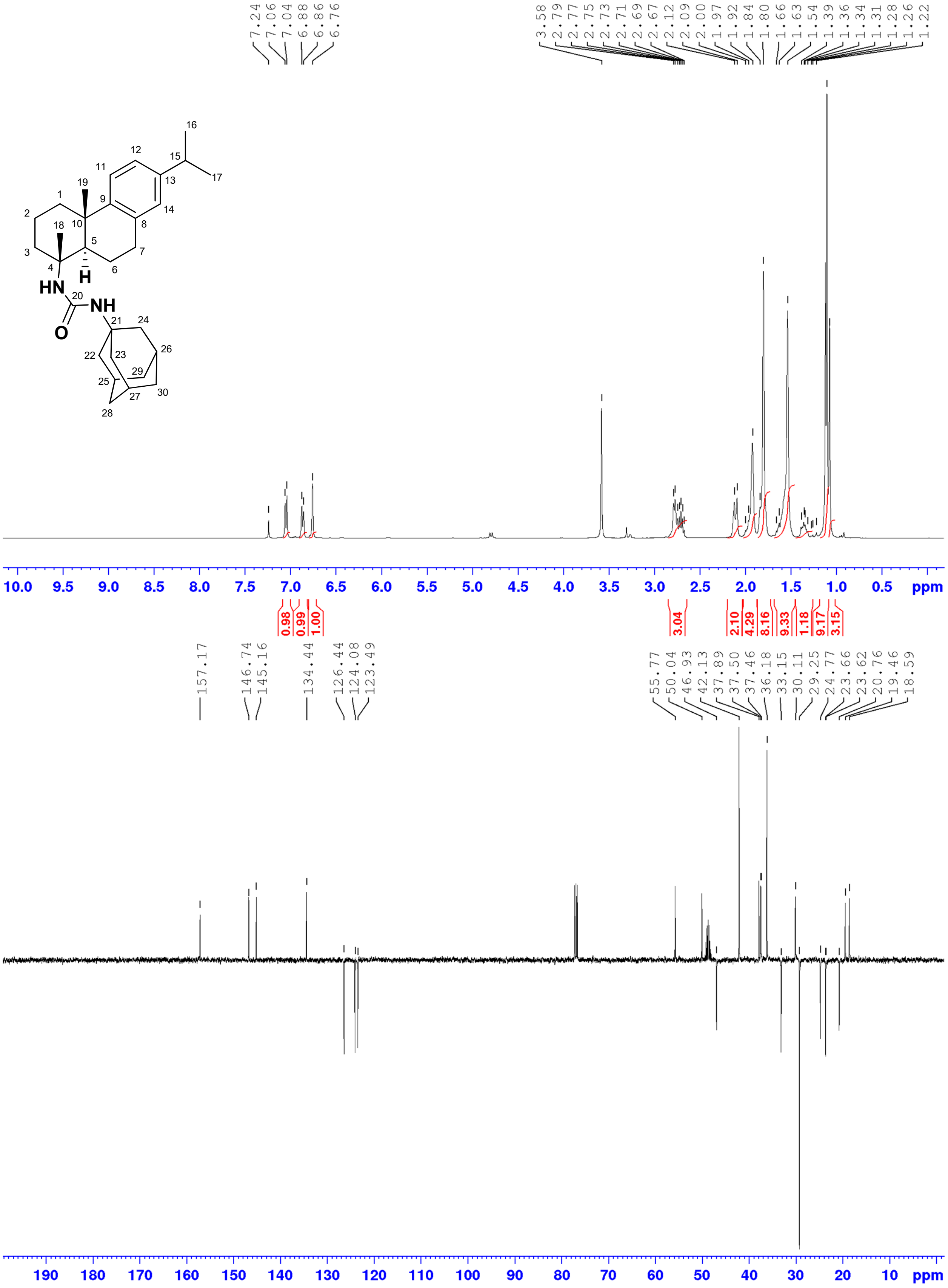

^{1}H and ^{13}C NMR spectra for compound 10

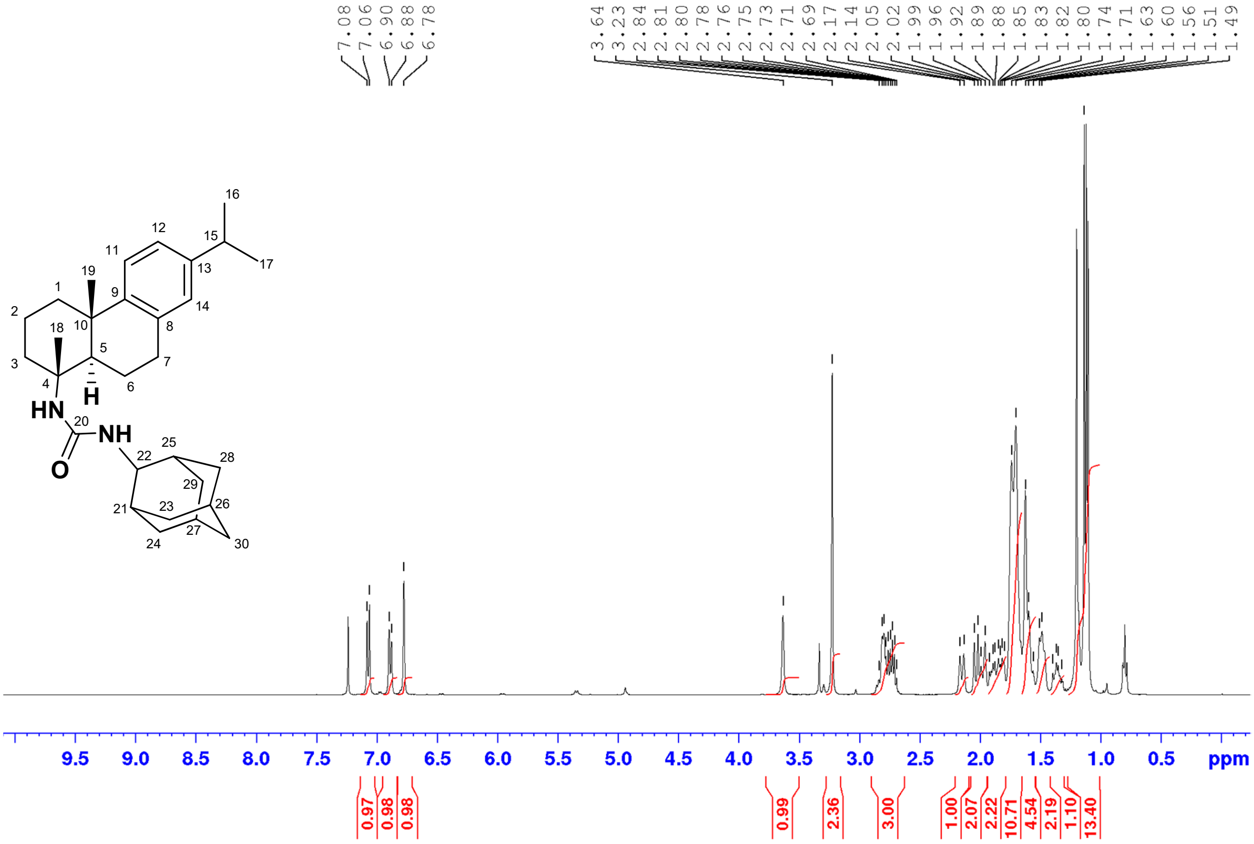

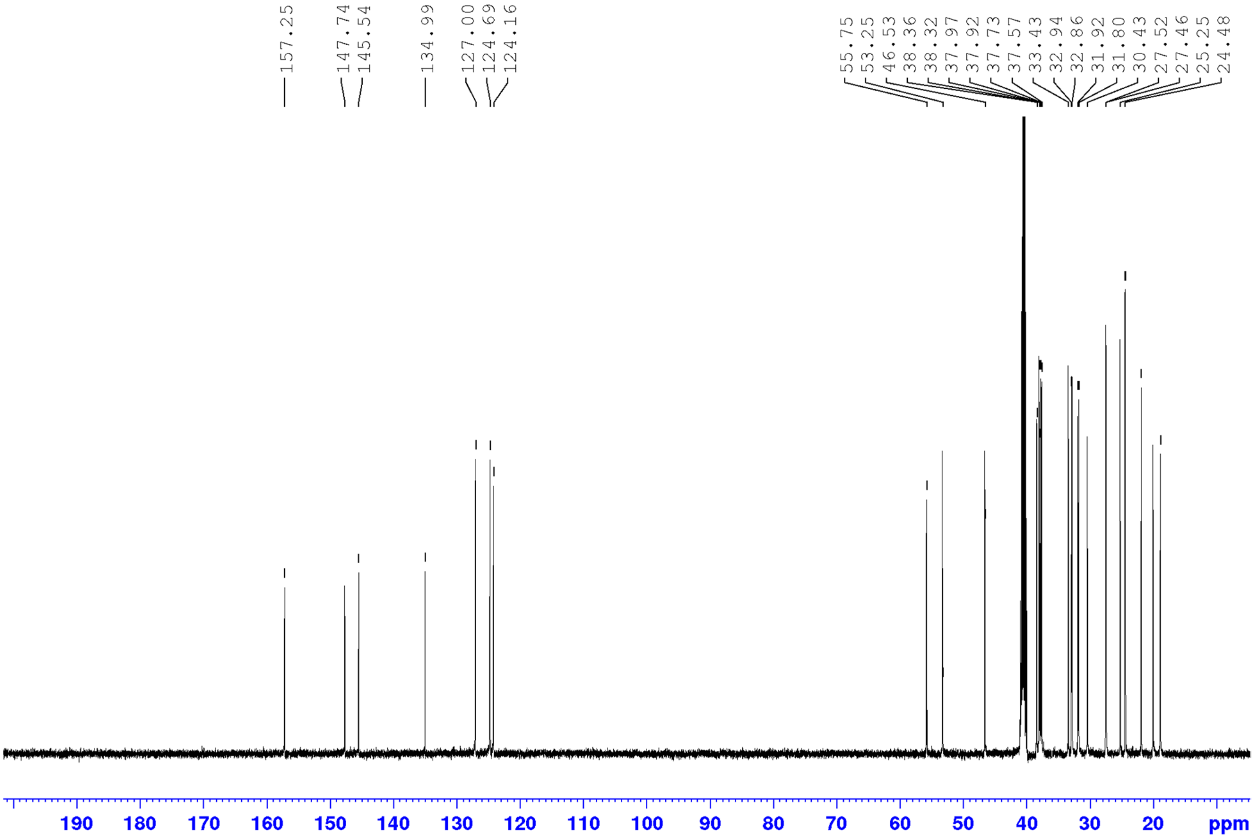

^{1}H and ^{13}C NMR spectra for compound 11

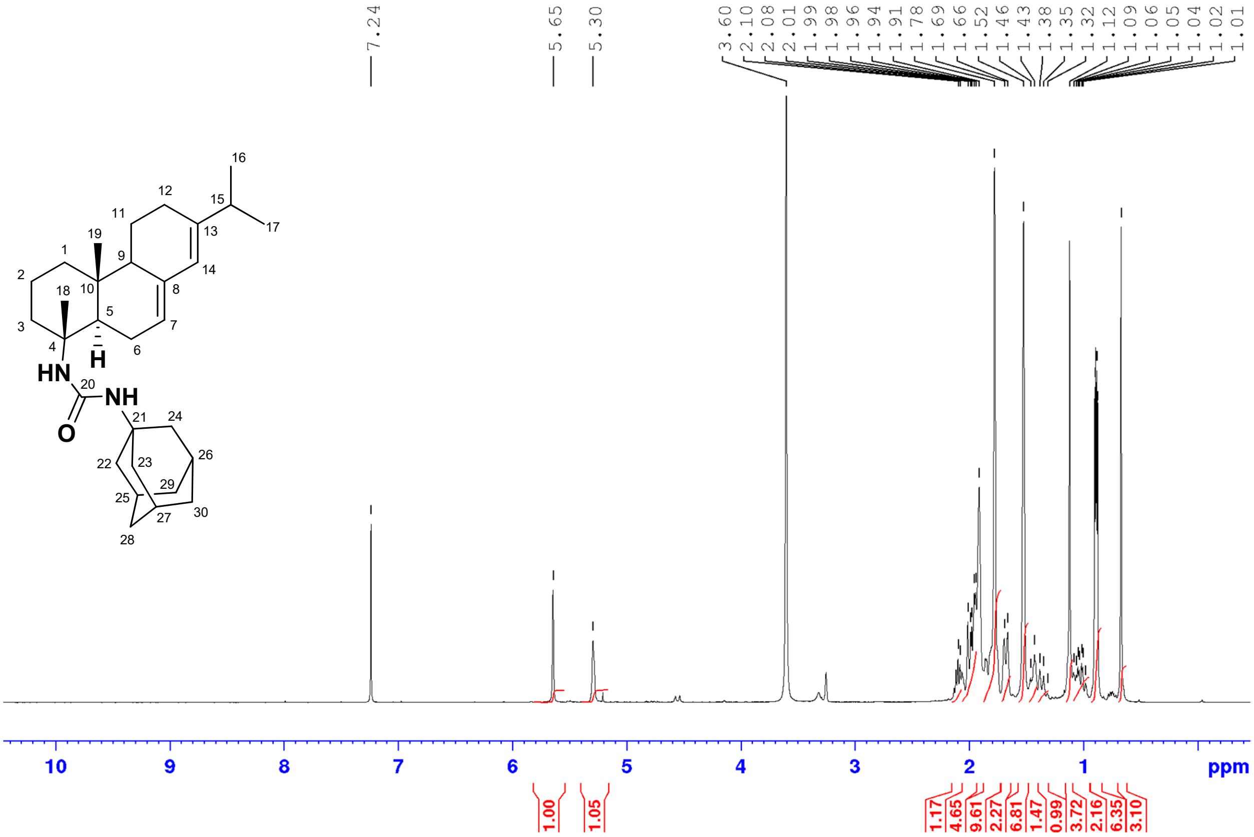

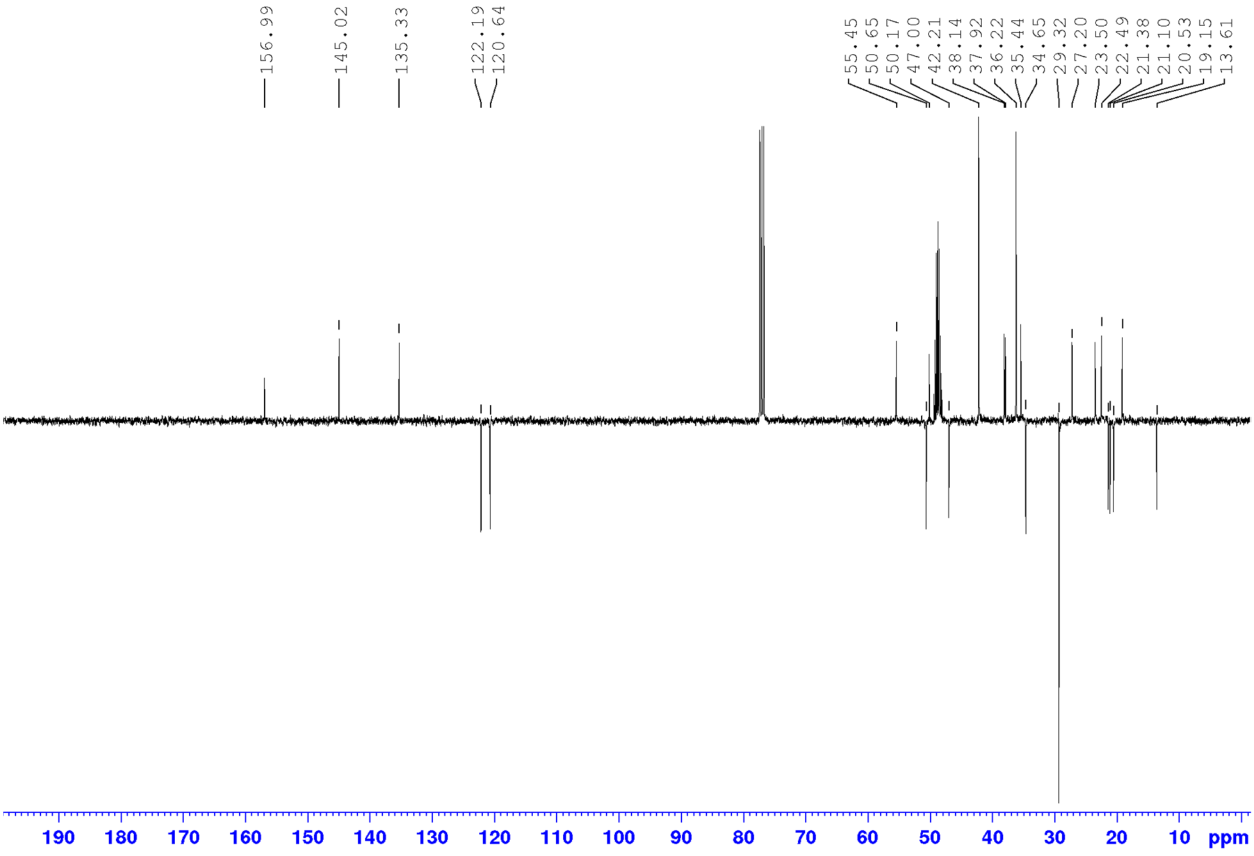

^{1}H and ^{13}C NMR spectra for compound 12

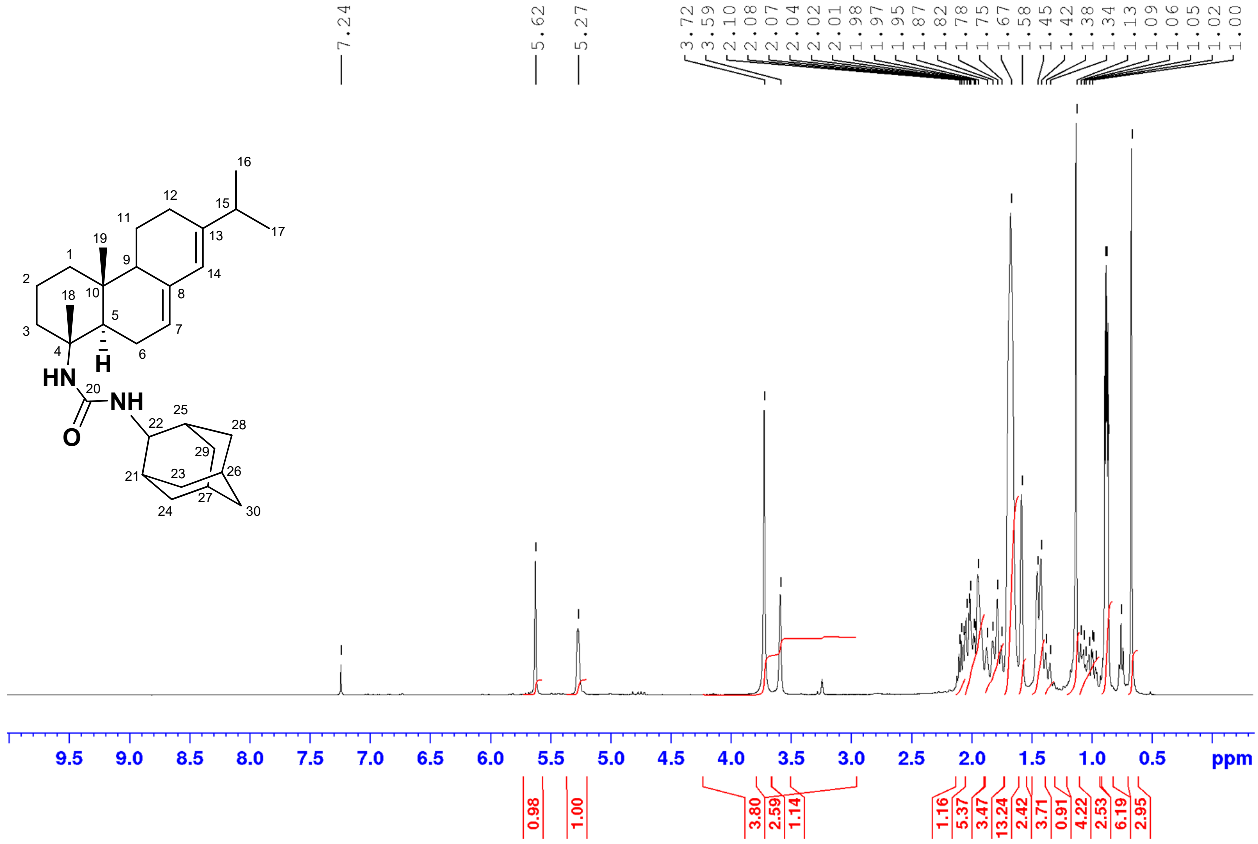
7.24
5.62
5.27
3.72 3.59 2.10 2.08 2.07 2.04 2.02 2.01 1.98 1.97 1.95 1.87 1.82 1.78 1.75 1.67 1.58 1.45 1.42 1.38 1.34 1.13 1.09 1.06 1.05 1.02 1.00
16 12 15 11 19 17 9 13 1 2 18 10 14 8 5 3 4 7 6
H
HN 20 NH
O
22 25 28 29 21 23 26 24 27 30
0.98 1.00 3.80 2.59 1.14 1.16 5.37 3.47 13.24 2.42 3.71 0.91 4.22 2.53 6.19 2.95
9.5 9.0 8.5 8.0 7.5 7.0 6.5 6.0 5.5 5.0 4.5 4.0 3.5 3.0 2.5 2.0 1.5 1.0 0.5 ppm

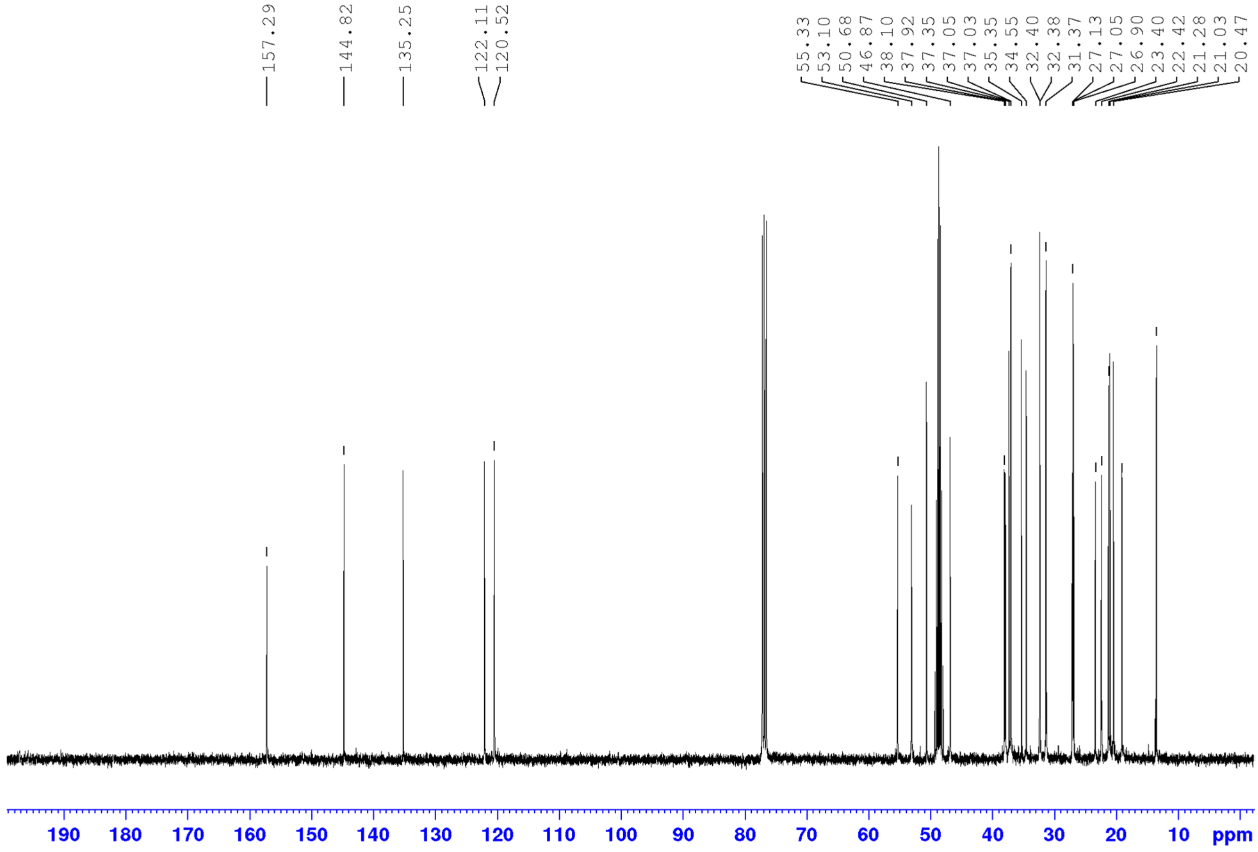
157.29
144.82
135.25
122.11 120.52
55.33 53.10 50.68 46.87 38.10 37.92 37.35 37.05 37.03 35.35 34.55 32.40 32.38 31.37 27.13 27.05 26.90 23.40 22.42 21.28 21.03 20.47
190 180 170 160 150 140 130 120 110 100 90 80 70 60 50 40 30 20 10 ppm

Table S1. LogP values for adamantane derivatives predicted with GALAS algorithm (implemented in the ACD/Percepta software, www.acdlabs.com), and the corresponding rate of brain penetration (LogPS) and extent of brain penetration (LogBB) calculated using LogP, molecular size, and H-bonding parameters as inputs. LogPS varies from -2.2 to -1.4 and LogBB from 0.2 to 0.48, which is suitable for penetrating into the central nervous system.

Compound	$LogP^{GALAS}$	LogPS	LogBB
		-5 Almost impermeable; -3 Average permeability; -1 Freely diffusible	-1.5 Restricted to plasma; 0 Evenly distributed; 1.5 Concentrated in brain tissue
1	7.50	-2.1	0.24
2	7.31	-1.9	0.20
3	7.60	-2.0	0.30
4	7.38	-1.9	0.27
6	7.48	-1.8	0.48
7	7.34	-1.7	0.48
8	8.28	-2.2	0.29
9	7.19	-1.8	0.30
10	7.27	-1.9	0.29
11	6.72	-1.6	0.47
12	6.30	-1.4	0.29
